# Comparative Evaluation of the Shear Bond Strength of Composite to Remineralized Dentin: An In Vitro Study

**DOI:** 10.7759/cureus.92118

**Published:** 2025-09-12

**Authors:** Rucha Pawar, Roshan M Samuel, Priyanka Zinge, Aayush P Bhatt, Savani Purohit

**Affiliations:** 1 Department of Conservative Dentistry and Endodontics, School of Dental Sciences, Krishna Vishwa Vidyapeeth (Deemed to be University), Karad, IND

**Keywords:** composite resin, nanohydroxyapatite, self-assembling peptide, shear bond strength, silver diamine fluoride (sdf)

## Abstract

Background

Caries-affected dentin is a partially demineralized substrate that retains its collagen matrix and has the potential to be remineralized. Recent biomimetic materials have shown potential in enhancing remineralization and improving composite bonding. The study aimed to compare the shear bond strength of composite resin to demineralized dentin after remineralization with three agents: silver diamine fluoride (SDF), nanohydroxyapatite (nHAp), and self-assembling peptide P11-4 (SAP P11-4).

Materials and methods

An in vitro, non-randomized, experimental study was conducted using sixty premolars with a single root, collected post-extraction, and equally divided into four groups: control, SDF, nHAp, and SAP. Remineralizing agents were applied to exposed dentin surfaces, followed by the placement of composite resin. After surface treatment with remineralizing agents, the resin-based composite was applied, and a universal testing machine was utilized to determine the shear bond strength. The modes of failure were examined using a stereomicroscope. One-way ANOVA and Tukey’s post hoc test were used (p < 0.001).

Results

The SAP P11-4 group showed the highest mean bond strength (28.13 N), followed by the SDF groups and nHAP groups, while the control group demonstrated the lowest values. All remineralized groups exhibited significantly higher bond strength compared to the control (p < 0.001). Intergroup comparisons revealed statistically significant differences among all test groups, with SAP P11-4 significantly outperforming SDF and nHAP. Failure mode analysis revealed that SAP P11-4 predominantly resulted in cohesive failure, whereas SDF and nHAP primarily exhibited adhesive failure.

Conclusions

Among the three remineralizing agents tested, SAP P11-4 demonstrated the highest shear bond strength to demineralized dentin. Its superior performance highlights its potential to enhance the longevity and durability of resin-based restorations in minimally invasive dentistry. The predominance of cohesive failure further supports its effectiveness in creating a strong, integrated bond between the composite resin and dentin substrate.

## Introduction

For the treatment of cavitated dentin lesions, minimally invasive techniques form the foundation of modern restorative dentistry concepts. It is sufficient to eliminate the denatured caries-infected dentin layer, which is heavily contaminated by bacteria, to guarantee caries arrest. Caries-affected dentin (CAD), the inner layer, is partially demineralized but amenable to remineralization [1]. The uneven demineralization of CAD limits resin monomers from penetrating exposed collagen fibrils, which leads to water buildup along the bonded interface that is vulnerable to hydrolytic breakdown [2]. To enhance the mechanical and bonding qualities of the partially demineralized dentin, emerging methods were used that focus on remineralization [3].

Since the early 1970s, silver diamine fluoride (SDF) has been used worldwide to control dental caries, primarily in pediatric patients [4]. It has the chemical formula Ag(NH₃)₂F and exists as a colorless aqueous solution containing ions. Silver combats cariogenic bacteria, while fluoride promotes tooth remineralization [4]. Another material used is nano-hydroxyapatite (nHAp). Hydroxyapatite (HA) is composed of calcium and phosphate ions, with the formula Ca₁₀(PO₄)₆(OH)₂ and a calcium-to-phosphorus ratio of approximately 1.67 [5]. The primary ingredient of enamel is HA, which gives the surface a white appearance [6]. A very recent and successful method for reversing early caries lesions is employing self-assembling peptide (SAP) P11-4. Upon contact with the tooth structure, the SAP solution infiltrates the lesion. P11-4 works well for treating early caries lesions when used once [7].

Therefore, this study aimed to evaluate and compare the effects of three biomimetic remineralizing agents, namely SDF, nanohydroxyapatite (nHAp), and self-assembling peptide P11-4 (SAP P11-4), on the shear bond strength of composite resin to demineralized dentin, using an etch-and-rinse adhesive protocol. The study also examined the failure modes following bonding. The null hypothesis tested was that there would be no significant difference in the shear bond strength of composite resin to demineralized dentin pre-treated with SDF, nHAp, or SAP P11-4.

## Materials and methods

The study proceeded after procuring ethical committee approval (IEC approval no. KIMSDU/IEC/06/2023). This in vitro, non-randomized experimental study was designed to compare the bond strength of resin-based composite to dentin treated with different remineralizing agents. According to the convenience sampling scheme, 60 extracted human premolars with single roots were collected for this in vitro study. The sample size was determined using G*Power software (version 3.0.1, Heinrich-Heine-Universität Düsseldorf, Germany). A total of 60 specimens (15 per group across four groups) was estimated as the minimum required to achieve 80% statistical power, with an assumed effect size of 0.45 and a significance level of 0.05. This calculation was based on data trends observed in a previous study by Alagha [8], which evaluated the micro-shear bond strength of nanohybrid composite resin following treatment with different remineralizing agents.

A cylindrical plastic mold, measuring 15 mm in diameter and 20 mm in height, was filled with self-curing acrylic resin. A separating medium was applied to the mold, and to ensure a flat and smooth base, the mold was positioned on a glass slab. The teeth were vertically implanted, exposing the occlusal surface above the mold surface and reaching the cementoenamel level. Enamel on the occlusal surface was ground off using a diamond disc until uniform dentin exposure was achieved across all samples.

To get rid of any remaining debris, the freshly sliced dentin surfaces were thoroughly washed with water. All surfaces except the occlusal were protected using nail polish, creating a barrier against shear force transmission. Remineralizing gel was applied to the freshly exposed dentin using a microbrush. 60 freshly extracted premolars were stored in distilled water at room temperature to maintain hydration and preserve dentin integrity. The samples were randomly distributed into four groups (n = 15 each): Group I served as the control with no treatment; Group II received SDF; Group III was treated with nHAp; and Group IV received SAP P11-4. For Group I (Control), samples received no treatment and were dried using cotton pellets. For Group II, SDF (38% SDF, FAgamin^®^) was applied to dried dentin using a microbrush and left in place for three minutes. The specimens were subsequently immersed in artificial saliva for 30 minutes and then rinsed with water. For Group III, nHAp gel (99.5% purity, Ultrananotech Pvt. Ltd, Bangalore, India) was applied to dentin surfaces using a microbrush and allowed to remain on the dentin surfaces for two minutes each day over a period of five consecutive days, and specimens were kept at 37°C in artificial saliva. On the final day, after 15 seconds, the gel was gently washed off with distilled water, followed by air drying. For Group IV, SAP P11-4 (Curodont™ Repair) was applied following 20 seconds of 37% phosphoric acid etching (D-Tech Gel), as per the manufacturer’s instructions. The surface was then rinsed with distilled water, air-dried, and the agent was coated for three to five minutes using a microbrush.

The exposed occlusal dentin was etched for 15 seconds using 37% phosphoric acid (D-Tech Gel). The surface was flushed for 15 seconds with water and then gently air-dried. Bonding of the tooth surface of all samples was carried out using a universal bonding agent (3M Adper Single Bond, 3M Company, St. Paul, MN, USA). Bonding agent was applied in two layers, each for 15 seconds, then softly air-dried. Curing was done for 10 seconds.

Pediatric IV tubes that were 3 mm long and 1 mm in internal diameter were cut and utilized as molds to create nanohybrid composite specimens in order to standardize the use of composites. Following that, the tubes were laid on the dentin surface. Composite was placed in incremental layers (<2 mm thick). Each layer of composite was exposed to light curing for 20 seconds using an LED curing device (Woodpecker Light Cure Unit, China), with the device’s tip held approximately 2 mm away from the surface. After curing, the tubes were then removed to obtain cylinders of composite.

**Figure 1:**
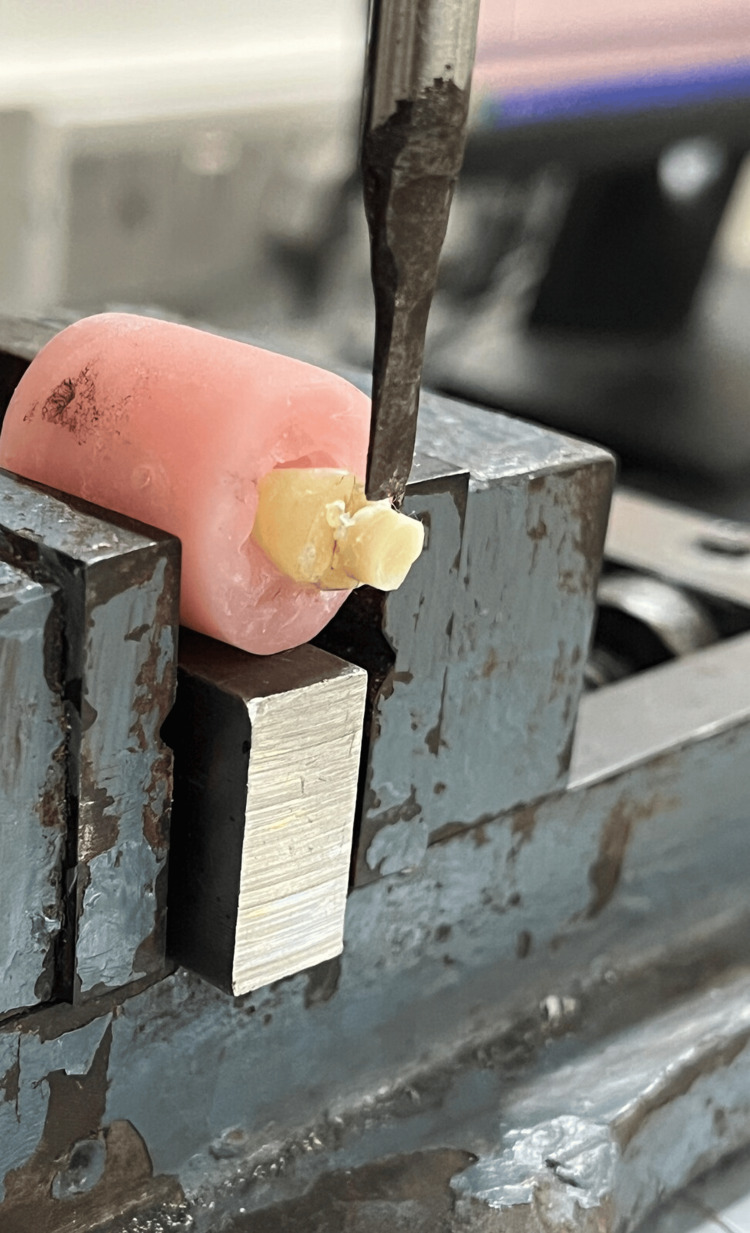
Mold placed on universal testing machine

Each mold was secured with screws to the Universal Testing Machine (Series 6800, Instron^®^ Corp., Norwood, MA, USA), which was then used to apply the shearing load (Figure 1), followed by stereomicroscopic examination of the fractured specimens.

For bond strength testing, a force of 25 MPa was applied at 0.5 mm/min crosshead speed. The loading was continued until bond failure was observed.

Following tests for shear bond strength, the specimens were observed under a stereomicroscope. Observations were conducted using a stereomicroscope at a magnification of 25X. Under the stereomicroscope, the failure mode was assessed and categorized into one of the following types: adhesive failure, which occurs at the adhesive-substrate junction; cohesive failure, which takes place within the bonded materials themselves; and mixed failure, which exhibits a combination of both adhesive and cohesive characteristics.

This study utilized a non-randomized convenience sample based on specimen availability, which may limit the generalizability of the findings. However, efforts were made to minimize variability by standardizing the specimen preparation and testing protocols. All specimens were prepared by a single calibrated operator to ensure consistency. Treatment allocation was performed by a second, blinded investigator using sealed opaque envelopes. While partial blinding of the operator was implemented during shear bond strength testing, complete blinding was not feasible due to the characteristic dark staining of the SDF-treated specimens. As with all in vitro studies, these methodological constraints should be considered when interpreting the results and extrapolating them to clinical practice.

Statistical analysis

Statistical evaluations were conducted via IBM SPSS Statistics for Windows, Version 21.0 (Released 2012; IBM Corp., Armonk, NY, USA). Quantitative data were summarized and expressed as mean values accompanied by their respective standard deviations. A confidence interval of 95% was established, with the level of statistical significance set at 0.05. A statistical power of 80% was intended for the investigation in order to identify significant differences. The general comparison of the four experimental groups was done using one-way ANOVA. Following this, Tukey’s post hoc multiple comparison test was applied to identify pairwise differences between groups, with adjustments made for multiple comparisons.

## Results

**Figure 2:**
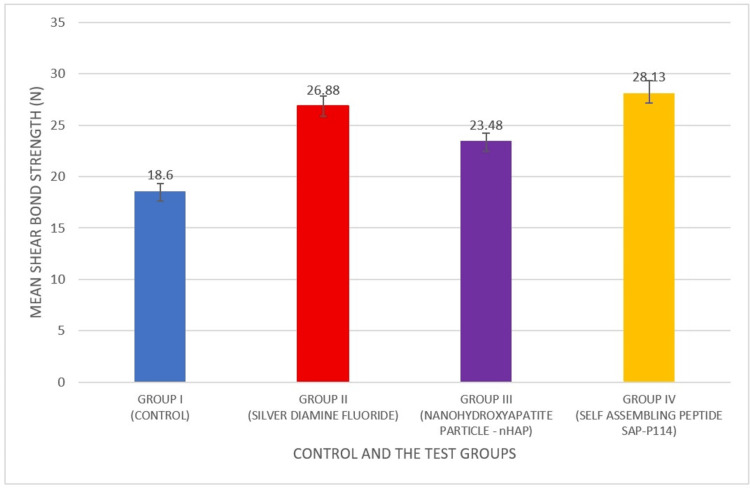
Impact of various remineralizing agents on the bonding strength of dental composites

Figure 2 shows that Group I exhibited the lowest mean shear bond strength (18.60 N, SD = 0.73), while the highest was recorded in Group IV (28.13 N, SD = 1.20). The overall comparison revealed a highly significant difference among the groups (F = 314.675, p < 0.001).

Table 1 presents the intergroup comparisons, showing statistically significant differences between each group, as determined by Tukey’s honestly significant difference post hoc analysis.

**Table 1:** Intergroup comparison of the shear bond strength of the composite using Tukey’s honestly significant difference test ^*^ p < 0.05 indicates a statistically significant difference. ^**^ p < 0.001 indicates a highly statistically significant difference. nHAp, nanohydroxyapatite; SAP P11-4, self-assembling peptide P11-4; SDF, silver diamine fluoride

Intergroup comparison	Mean difference	Standard error	p-Value	95% CI lower bound	95% CI upper bound
Group I (Control) vs. Group II (nHAp)	-8.2867	0.33994	<0.001^**^	-9.1868	-7.3865
Group I (Control) vs. Group III (SDF)	-4.88	0.33994	<0.001^**^	-5.7801	-3.9799
Group I (Control) vs. Group IV (SAP P11-4)	-9.5333	0.33994	<0.001^**^	-10.434	-8.6332
Group II (nHAp) vs. Group III (SDF)	3.40667	0.33994	<0.001^**^	4.3068	4.3068
Group II (nHAp) vs. Group IV (SAP P11-4)	-1.2467	0.33994	0.003^*^	-0.3465	-0.3465
Group II (SDF) vs. Group IV (SAP P11-4)	-4.6533	0.33994	<0.001^**^	-5.5535	-3.7532

Comparing Group I to Groups II, III, and IV, the bond strength was significantly lower (p < 0.001 for all). Group III showed a considerably stronger relationship than Group IV (p = 0.003), but not as strong as Group II (p < 0.001). Furthermore, Group IV had a substantially stronger relationship than Group II (p < 0.001). These results reveal that shear bond strength was greatly increased by all remineralizing agents relative to the control group, with the SAP P11-4 group exhibiting the largest improvement.

In terms of failure modes observed under stereomicroscopy, Groups II and III predominantly exhibited adhesive failure, showing a clean separation at the interface between two different materials, typically between a bonded layer and a core/substrate, which was seen, suggesting a weak bond at the dentin-composite interface. Group I displayed mixed-mode failure, as there was no clean separation along the entire interface, and both the bonded interface and internal areas of the material showed signs of fracture, indicating overall poor bond strength. Group IV exhibited cohesive failure; the margin was intact, and there was no evidence of clean separation at the interface between the bonded material and the core, implying that the bond was stronger than the internal strength of the composite material.

## Discussion

This study aimed to directly compare the effects of three remineralizing agents, such as SDF, nHAp, and SAP P11-4, on composite bonding to CAD under standardized conditions. Unlike previous studies that evaluated individual agents in isolation, this comparative approach addresses a gap in the literature by providing a side-by-side analysis of bond strength and failure modes. The findings are particularly relevant in the context of biomimetic and minimally invasive dentistry, offering insights into the integration of these agents with dentin substrates to support more durable restorations.

The objective of a fully integrated tooth-restoration junction is crucial but rarely accomplished. Micro gaps at the tooth-restoration junction and irregularities at the cavosurface margin are commonly present, promoting bacterial infiltration and contributing to secondary caries development. Considering this, the concept of restorative tooth surface junction remineralization is established [9].

Fluoride varnishes have mostly been utilized to treat the initial enamel or white spot lesions for the reversal of the lesions, with promising results. However, due to certain limitations, the quest to develop newer and more advanced materials has resulted in the creation of novel materials [10,11].

SDF exhibits anticariogenic effects primarily because of the combined action of silver and fluoride ions, which exert both antibacterial and remineralizing effects on dental tissues. When SDF reacts with water and other biological fluids, it liberates silver ions, which have numerous modes of action that target different biological organisms, their subcellular targets, and metabolic systems [12].

High levels of fluoride attach to bacterial cell components and prevent the production of biofilms [13]. The bond strength was comparable between the control and SDF-treated groups, exhibiting no significant variation and showing adhesive failure, which is the leading cause of most failures. SDF contamination of the surface may cause a decrease in bond strength [14]. An in vitro investigation shows that the addition of potassium iodide with SDF (SDF-KI) and chlorhexidine is effective against *Streptococcus mutans* [15]. According to Siqueira et al. [16], the application of diamine products enhanced the micro tensile bond strength, but SDF shouldn't be applied over the entire tooth surface. According to Yadav et al. [17], SDF showed the best remineralizing potential in energy dispersive X-ray and scanning electron microscopy. Fluoride toothpaste, fluoride-free toothpaste, casein phosphopeptide-amorphous calcium fluoride phosphate, and casein phosphopeptide-amorphous calcium phosphate (CPP-ACP) were the next best alternatives. Applying SDF greatly increased Giomer's binding strength with carious dentin when compared to Activa BioActive, which makes it an excellent option for restoring posterior teeth areas [18].

nHAp (Ca₁₀(PO₄)₆(OH)₂) shares structural similarities with the primary mineral found in bone and teeth. Its effectiveness can be further increased by products that contain nanoparticles, which can improve the precipitation of the phosphate and calcium ions in the tooth structure [19]. nHAp primarily inhibits dental cavities by increasing the surface area of the proteins that bind to it. Because of its small size, it fills in the enamel surface depression and restores it. Using smaller nHAp particles with heterogeneous rod architectures was suggested to improve the surface area that is accessible for mineral attachment and release. Additionally, it increases the adhesive’s resistance to deterioration by lowering stress contraction, enzymatic collagen degradation, and hydrophilicity [20]. After remineralization, the mean microhardness differs significantly. In descending order, the mean value was highest for fluoride, CPP-ACP, bioactive glass, and nHAp [21].

SAP is known to spontaneously assemble into organized nanostructures. It was first demonstrated that peptides with the ability to self-assemble efficiently serve as building blocks for an array of materials and device applications. As for surface microhardness and the depth of remineralization, or absolute depth profile, SAPs have demonstrated encouraging outcomes [22]. This finding demonstrates the effectiveness of SAPs in reversing early enamel lesions or white spot lesions. Self-assembly of P11-4 is driven by electrostatic interactions between cationic arginine and anionic glutamic acid residues. Four negatively charged Glu residues found in P11-4, which is organized into fibers, may represent Ca2+ binding sites [23]. Remineralization of undamaged and demineralized enamel is possible with SAP P11-4 and calcium silicate and sodium phosphate. Increased remineralization was seen in demineralized samples that were exposed to erosion [24].

In this study, SAP P11-4 demonstrated promising outcomes by significantly enhancing shear bond strength and influencing the failure pattern with composite resin, outperforming both SDF and nHAp. While nHAp is biomimetic and biocompatible, it primarily supports passive remineralization by depositing mineral particles onto the dentin surface. This process tends to form a superficial layer that is less integrated with the underlying dentin. In contrast, SAP P11-4 promotes biological integration with the dentin substrate by forming a structurally sound, mineral-rich layer that preserves permeability and microstructure, which are key factors for optimal adhesive penetration.

This in vitro study has certain limitations that should be acknowledged when interpreting the results. First, a non-randomized convenience sampling method was used due to the availability of extracted premolars, which may affect the generalizability of the findings. Although efforts were made to standardize all specimen preparation and testing procedures, biological variability among teeth cannot be entirely eliminated. Second, partial operator blinding was implemented during shear bond strength testing. While the treatment allocator was blinded using sealed envelopes, complete blinding of the operator was not possible due to the characteristic black staining of specimens treated with SDF, potentially introducing detection bias. Finally, as with all in vitro studies, the absence of intraoral conditions such as saliva, temperature fluctuations, and occlusal forces limits the ability to directly extrapolate the findings to clinical settings. Further in vivo studies with larger, randomized samples are recommended to validate these results.

## Conclusions

This study contributes novel comparative data supporting the use of SAP P11-4 to enhance composite bonding to CAD, with implications for improved clinical outcomes in restorative dentistry. Treatment with SAP P11-4 (Curodont™ Repair) significantly increased the shear bond strength of dental composite resin bonded to demineralized dentin, outperforming both nHAp and SDF.

Analysis of failure modes further highlighted the superiority of SAP P11-4. Adhesive failures were most commonly observed in the nHAp and SDF groups, while the control group primarily exhibited a mixed failure pattern. In contrast, the SAP P11-4 group predominantly demonstrated cohesive failures, indicating the formation of a much stronger and more durable bond between the composite and the dentin substrate.
